# Virulence of viral haemorrhagic septicaemia virus (VHSV) genotype III in rainbow trout

**DOI:** 10.1186/s13567-015-0303-z

**Published:** 2016-01-08

**Authors:** Takafumi Ito, Jun Kurita, Koh-ichiro Mori, Niels J. Olesen

**Affiliations:** Tamaki Laboratory, Aquatic Animal Health Division, National Research Institute of Aquaculture, Fisheries Research Agency, 224-1 Hiruda, Tamaki, Mie Japan; Aquatic Animal Health Division, National Research Institute of Aquaculture, Fisheries Research Agency, 422-1 Nakatsuhamaura, Minami-Ise, Mie Japan; National Veterinary Institute, Technical University of Denmark, Bülowsvej 27, 1870 Frederiksberg C, Denmark

## Abstract

In general, viral haemorrhagic septicaemia virus (VHSV) isolates from marine fish species in European waters (genotypes GIb, GII and GIII) are non- to low virulent in rainbow trout. However, a VHSV isolation was made in 2007 from a disease outbreak in sea farmed rainbow trout in Norway. The isolate, named NO-2007-50-385, was demonstrated to belong to GIII. This isolate has attracted attention to assess which of the viral genome/proteins might be associated with the virulence in rainbow trout. In this study, we describe the difference of virulence in rainbow trout between the NO-2007-50-385 and 4p168 isolates as representatives of virulent and non-virulent GIII isolates, respectively. Rainbow trout were bath challenged with VHSV NO-2007-50-385 for 1 and 6 h, resulting in cumulative mortalities of 5 and 35%, respectively. No mortality was observed in the rainbow trout groups immersed with the genotype III VHSV isolate 4p168 for 1 and 6 h. The viral titre in organs from fish challenged with NO-2007-50-385 for 6 h increased more rapidly than those exposed for 1 h. By in vitro studies it was demonstrated that the final titres of VHSV DK-3592B (GI), NO-2007-50-385 and 4p168 inoculated on EPC cells were very similar, whereas when inoculated on the rainbow trout cell line RTG-2 the titre of the non-virulent 4p168 isolate was 3–4 logs below the two other VHSV isolates. Based on a comparative analysis of the entire genome of the genotype III isolates, we suggest that substitutions of amino acids in positions 118–123 of the nucleo-protein are candidates for being related to virulence of VHSV GIII in rainbow trout.

**Electronic supplementary material:**

The online version of this article (doi:10.1186/s13567-015-0303-z) contains supplementary material, which is available to authorized users.

## Introduction

Viral haemorrhagic septicaemia virus (VHSV) is known as the causative agent of serious diseases occurring in wild and farmed fish in the Northern Hemisphere. Until the beginning of the 1990’s the disease was believed to cause severe mortalities only in farmed rainbow trout in Continental Europe. In the last three decades, however, VHSV has been isolated from more than 80 fresh- and seawater fish species in North America, North East Asia and Europe [1–4].

VHSV isolates can be divided into four major genotypes and a number of subtypes with rather distinct geographical distributions [5–7]. The host range and the pathogenicity appear, at least to some extent, to be linked to the genotype. In general, VHSV isolated from marine fishes (genotypes GIb, GII, GIII and GIVa) are non- or low virulent for rainbow trout *Oncorhynchus mykiss* [8]. Several research groups have searched for traits determining virulence mechanisms of VHSV [9–11]. Béarzotti et al. reported that the glycoprotein (G)-protein seemed to play an important role in the virulence to rainbow trout [9]. Snow and Cunningham reported that the virulence by rainbow trout of the VHSV GIII isolate UK-860/94, which was isolated from an outbreak in farmed turbot, increased after five in vivo passages [10]. In this study, the viral G-gene sequences were examined for all five passages, resulting in 100% similarity. From the results of comparison of the virulent isolates DK-Hededam (GI) and FR-14-58 (GIa) with the two non-virulent GIb isolates, UK-96-43 and Cod ulcus virus (synonym: M Rhabdo), Betts and Stone [11] suggested that only a limited number of changes in various viral proteins may be involved in whether an isolate becomes pathogenic or not. However, identification of which of the amino acid (aa) substitutions play the most important role for VHSV virulence remains elusive in rainbow trout. Since the viral G-protein is responsible for the production of neutralizing antibodies in fish [12–14], and has been suggested to play an important role in determining the virulence of VHSV [9, 15], specific mutations in the G-protein have been considered interesting. However, in a recent report, Kim et al. [16] showed that a substitution at position 1012 of the large polymerase (l)-protein of VHSV can change the virulence to rainbow trout gill epithelial cells. In addition Einer-Jensen et al. reported that differences in virulence among phylogenetically distinct isolates of VHSV are not explained by variability of the G-protein or the non-virion (Nv) protein [17]. These reports suggest that other viral proteins than the G- and Nv-protein may have a role in determining the virulence of VHSV in rainbow trout.

Stone et al. [15] argued that marine isolates of VHSV are a potential threat to the fish farming industry if the opportunity to adapt under intensive farming conditions is provided. An isolation of VHSV was made in 2007 from a disease outbreak in sea farmed rainbow trout in Norway. The isolate, named NO-2007-50-385, was demonstrated to belong to GIII [18]. Since in general GIII isolates have been found to be non- or having only very low virulence to rainbow trout [8], this isolate has attracted attention in order to assess which of the viral genome or proteins might be associated with the induction of virulence in rainbow trout. Duesund et al. [19] reported the full genome sequence of the virulent VHSV GIII isolate FA281107 (which is equivalent to NO-2007-50-385 in the Fish Pathogens Database [20]), and they concluded that the lack of information about the entire genome of the low virulent VHSV GIII isolates is a major problem for the study of the virulence factors of this virus.

In our previous studies, VHSV genotype specific mAbs were produced in order to establish a fast and low cost system for genotyping VHSV isolates [21, 22]. In the process, we encountered several unpredicted reactions. The VHS-3.75 mAb, reacts with all genotype III isolates except with the rainbow trout pathogenic isolate NO-2007-50-385 (from the Norwegian west coast) [18], and additionally reacted with the GIVc isolate, CA-NB00-01, (from the American east coast, New Brunswick) [23]. By aa alignment of the nucleo (*N*)-protein, the epitope of this mAb was found to include amino acids at positions 103, 118 and 121 [22]. The atypical reaction of this mAb could thus be related to the virulence of the isolate to rainbow trout. There was, however, no sequence information on the entire genome of VHSV GIII isolates with low virulence to rainbow trout, and thereby this epitopic part of the mAb VHS-3.75 could not be determined as the only domain responsible for virulence of VHSV GIII isolates in rainbow trout.

In this study, we describe the differences of pathogenicity in rainbow trout between the virulent NO-2007-50-385 and the non-virulent 4p168 VHSV GIII isolates. In addition, the few differences in the nucleotide composition of the full genome of the isolates are described and discussed. The resulting alignments indicate that substitutions of the 3 aa in the *N*-protein can be related virulence of VHSV GIII in rainbow trout. Moreover, we demonstrated that the non-virulent 4p168 isolate replicated to a lower titre in a rainbow trout cell line when compared to virulent isolates and to cell lines from other fish species, and we documented that the duration of exposure is crucial when describing virulence in infection by immersion trials.

## Materials and methods

### Virus and cell lines

The VHSV GIII isolates used for the present study were NO-2007-50-385 [18] and 4p168 [24], representing a rainbow trout virulent and a non-virulent isolate, respectively. In addition, the very high virulent rainbow trout VHSV Ia isolate DK-3592B was used as a positive control [25].

The bluegill fry (BF-2) cell line [26] was used for propagation, re-isolation and titration of the virus in organs from infected fish. This cell line as well as the *Epithelioma papulosum cyprini* (EPC) [27] and the rainbow trout gonadal (RTG-2) [28] cell lines were used to test their susceptibility towards infection with the VHSV isolates. The cell lines were maintained in minimum essential medium (MEM; Mediatech) supplemented with 10% FBS (Equitech-Bio) and antibiotics (100 IU/mL penicillin and 100 μg/mL streptomycin). The cultivation of BF-2 and EPC cells was conducted at 25 °C, while the RTG-2 cells were cultivated at 20 °C. Each virus isolate was propagated in 75 cm^2^ flasks (Greiner Bio-One) at 15 °C. The viral supernatant was aliquoted into 3.6 mL cryo tubes (Nunc™, Thermo Scientific) and stored at −85 °C until use. Before the experimental infections, the virus in one of the aliquots was quantified by end-point titration in 96-well plates (Corning). Propagation, re-isolation and titration of all isolates were conducted at 15 °C.

### Fish

Rainbow trout for both experimental infections were bred from VHSV, IHNV and IPNV free broodstocks at the Tamaki Laboratory of the National Research Institute of Aquaculture (NRIA), Fisheries Research Agency. The obtained eggs were disinfected with iodophor (200 mg/L, 15 min) after fertilization. Fry of rainbow trout were fed commercial crumble diets (Ayutech, Marinetech, Japan) until 10–21 days after hatching and were subsequently fed commercial pellets (Saki-Hikari^®^, Kyorin, Japan). All stages were maintained in well water at approximately 15–16 °C to prevent any infection.

### Experimental infection

The experimental design for the two infection studies is shown in Table 1. In experimental infection #1, 200 rainbow trout were used (sizes are shown in the table). The rainbow trout were divided into 10 groups of 20 fish each. Rainbow trout in two groups (40 fish) were infected with the DK-3592B isolate as positive control; fish in one of the groups were intraperitoneally (i.p.) injected with 0.1 mL supernatant of crude virus (10^5.0^TCID_50_/fish); in the other group fish were infected by immersion in 10^5.0^ TCID_50_ DK-3592B/mL at 13 °C well water for 1 h. Three groups were infected with the NO-2007-50-385 isolate; one group was i.p. injected with 0.1 mL supernatant of crude virus (10^5.0^ TCID_50_/fish); the other two groups were bath infected by immersion in 10^5.0^ TCID_50_ NO-2007-50-385/mL water at 13 °C for 1 and 6 h, respectively. Likewise, three groups were infected with the VHSV 4p168 isolate. Two groups were used as non-infected negative controls. One group was i.p. injected with 0.1 mL supernatant from non-infected BF-2 cell cultures. The other group was immersed in cell culture supernatant diluted 1:1000 in well water for 1 h at 13 °C. All injected fish were anaesthetized using 2-phenoxyethanol diluted 1:1000 in well water. All fish groups were kept in 60 L tanks at 13.2 °C (13.0–13.8 °C) and fed a commercial diet once a day. Mortality was observed for 46 days. All dead and surviving fish were collected and all samples were tested on cell cultures and by reverse transcription-PCR (RT-PCR). Samples of kidney and spleen were pooled from each fish, while the brains were sampled individually.

**Table 1:** Experimental design, and the initial total length and body weight of rainbow trout used in this study.

Experiment	Group (genotype)	Treatment (infectious route/negative control)	No. of samples	Total length (cm)	Body weight (g)
1	DK-3592B (Ia)	Intraperitoneal injection	20	8.96 ± 0.94	9.61 ± 2.53
		Immersion 1 h	20	6.12 ± 0.79	2.79 ± 0.73
	NO-2007-50-385 (III)	Intraperitoneal injection	20	9.10 ± 0.78	9.00 ± 2.48
		Immersion 1 h	20	6.35 ± 0.88	2.91 ± 0.81
		Immersion 6 h	20	6.59 ± 0.85	3.20 ± 0.93
	4p168 (III)	Intraperitoneal injection	20	9.28 ± 0.44	9.12 ± 1.88
		Immersion 1 h	20	6.62 ± 0.64	3.24 ± 0.78
		Immersion 6 h	20	6.77 ± 0.67	3.33 ± 0.82
	Negative control	Intraperitoneal injection	20	9.52 ± 0.59	9.97 ± 1.74
		Immersion 1 h	20	6.75 ± 0.48	3.49 ± 0.55
2	DK-3592B (Ia)	Immersion 1 h (for mortality observation)	20	6.19 ± 0.64	3.14 ± 0.79
		Immersion 1 h (for sampling)	21	5.72 ± 0.47	2.17 ± 0.65
	NO-2007-50-385 (III)	Immersion 1 h (for mortality observation)	20	6.42 ± 1.20	3.51 ± 0.87
		Immersion 1 h (for sampling)	21	5.84 ± 0.63	2.25 ± 0.60
		Immersion 6 h (for mortality observation)	20	6.56 ± 0.76	3.22 ± 1.17
		Immersion 6 h (for sampling)	21	6.07 ± 0.64	2.63 ± 0.85
	4p168 (III)	Immersion 1 h (for mortality observation)	20	6.67 ± 0.46	3.44 ± 0.66
		Immersion 1 h (for sampling)	21	5.98 ± 0.68	2.40 ± 0.82
		Immersion 6 h (for mortality observation)	20	6.66 ± 0.48	3.17 ± 0.80
		Immersion 6 h (for sampling)	21	5.96 ± 0.89	2.42 ± 1.08
	Negative control	Immersion 6 h	20	6.57 ± 0.34	3.23 ± 0.46

In experimental infection #2, 225 rainbow trout were used (sizes are shown in Table 1). The fish were divided into 11 groups of 20–21 fish each. Forty-one rainbow trout (two groups) were infected with DK-3592B isolate; both groups were bath infected by immersion for 1 h in 13 °C water at a viral concentration of 10^5.0^ TCID_50_/mL. Eighty-two rainbow trout (four groups) were infected with the NO-2007-50-385 isolate; fish in two of the groups were bath infected by immersion for 1 h in 13 °C well water at a viral concentration of 10^5.0^ TCID_50_/mL; fish in the two other groups were immersed for 6 h in the same concentration of virus. Likewise, eighty-two rainbow trout (four groups) were infected by immersion with the 4p168 isolate for 1 and 6 h, respectively. The remaining group was immersed with supernatant from non-infected BF-2 cell cultures diluted 1:1000 in well water for 6 h. Samples for sequential determination of viral titres in the fish were collected from one of the two replicate tanks in all five infected groups. The other replicate tanks were used for observation of mortalities. All fish groups were kept in 60 L tanks at 13.7 °C (13.1–14.0 °C) and fed a commercial diet once a day, and mortality was observed for 29 days after infection. All pooled samples of kidneys and spleen, and brain from both dead and surviving fish in the tanks used for mortality observation were tested for VHSV on cell cultures and by RT-PCR. In addition, the gills, brain, heart and kidney from 3 fish from each tank used for retrieving samples were collected on days 1, 3, 5, 7 and 13 after viral exposure as also were organs from fish that died in these tanks during the trial. All samples were subjected to viral titrations on cell cultures. All fish experiments and the handling of fish were in accordance with “Guidelines for Animal Experimentation” of the National Research Institute of Aquaculture, Fisheries Research Agency, Japan. Details of the experimental design, average total length and body weight of fish in each group at the initial stage of the challenge are also shown in Table 1.

### Virus re-isolation and titration of organs from experimental fish

At the end of experiment #1 all fish were sampled for virological examination to detect the presence or absence of virus, whereas in experiment #2 only samples from 1 of the 2 replicates were collected (observation group). Whole fish were kept at −85 °C until further processing. After thawing, two samples were produced from each fish consisting of (1) kidney and spleen tissue, and (2) brain tissue. Homogenates of kidney/spleen and brain were prepared using approximately 50-times volume of MEM and filtered (0.45 μm). Then 100 and 10 μL of the each filtrate were inoculated onto subconfluent 1 day-old BF-2 cells in 24-well culture plates and incubated at 15 °C. The cells were regularly observed for cytopathic effect (CPE) over a period of 14 days. The observation of CPE was considered a VHSV positive result.

In cases where the amount of virus was quantified in individual organs, the gills, brains, hearts and kidneys were weighed, homogenized and then diluted 1:50 in MEM before filtration (0.45 μm). The titrations were performed in 96-well plates (Corning, NY, USA) on BF-2 cells, in 10-fold dilutions (four replicates per dilution, and incubated at 15 °C for 2 weeks). The results are given as TCID_50_/g organ. Samples that did not give CPE were shown as 0 (not detected). Positive samples with titres under the detection limit for titration was given as <10^1.3^ TCID_50_/g organ.

### Reverse transcription polymerase chain reaction (RT-PCR)

All fish, both infected and non-infected, were tested by RT-PCR. Total RNA was extracted from the samples using the TRIzol^®^ Reagent (Life Technologies) following the recommended protocol. The total RNA was dissolved in 100 μL DNase/RNase-free distilled water (Life Technologies) and stored at −85 °C until further processing. For the RT-PCR, the SuperScript^®^ One-Step RT-PCR System with Platinum^®^*Taq* (Life Technologies) was used. The RT-PCR was performed using the specific primers and the thermocycling profile for VHSV described in the chapter on VHS of the Manual of Diagnostic Tests for Aquatic Animals [29], with the exception that the volume was scaled-down from 50 to 20 μL.

### Statistical analysis

In experimental infections #1 and #2, the Fisher’s test was used to compare cumulative mortality between each infection group and negative control group. Statistical significance was determined, at *p* < 0.01.

### Multiplication of the three VHSV isolates in various cell lines

The multiplication in various cell lines of the three VHSV isolates (DK-3592B, NO-2007-50-385 and 4p168) was assessed by titration on BF-2, EPC and RTG-2 cell lines. Titration was performed by the endpoint dilution method, and the results expressed as TCID_50_/mL. To ensure experimental reproducibility, two batches (sample A and B) of each virus cell culture supernatant were used.

### Sequencing the 4p168 isolate

The viral genome of isolate 4p168 was sequenced as follows: the VHSV isolate was concentrated and sucrose gradient purified as described by Nishizawa et al. [30]. Viral RNA was extracted from the purified virus using the ISOGEN-LS^®^ (NIPPON GENE), and subjected to RT-PCR amplification with primers designed according to the sequence of strain FA281107 (GIII) (GenBank accession no. EU481506) for sequencing of each gene (Additional file 1). The entire nucleotide sequence of the coding region (from N to L gene) of the genome was determined by direct sequencing of each RT-PCR products. The 3′ termini was cloned using primer 5′-CTC GAT GAT GAT GAT GAT CTC-3′ [31] and the TOPO^®^ TA Cloning^®^ Kit (Life Technologies) with SURE 2 SuperCompetent Cells (Agilent Technologies). The 5′ termini was cloned using SMARTer™ RACE cDNA Amplification Kit (Clontech) and pGEM^®^-T Easy Vector Systems (Promega). For all nucleotide sequencing, BigDye^®^ Terminator v3.1 Cycle Sequencing Kit (Life Technologies) and ABI PRISM^®^ 3100 Genetic Analyzer (Life Technologies) were used.

## Results

### Infection experiment 1

**Figure 1:**
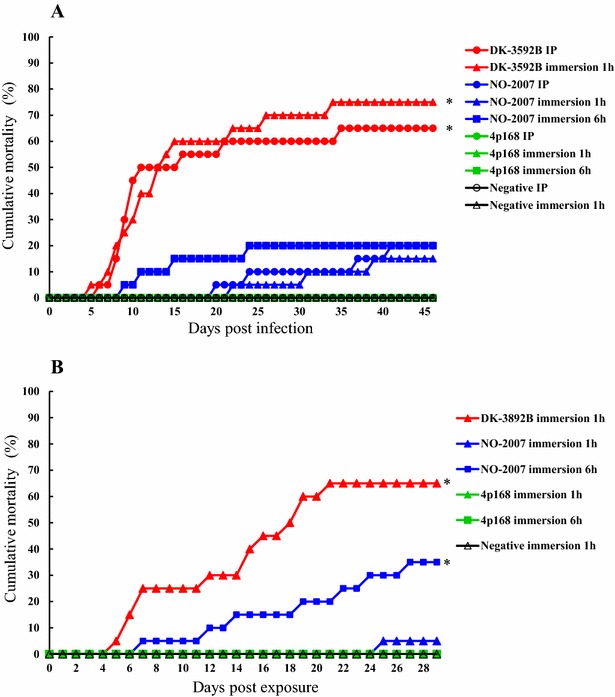
**Cumulative mortalities in experimental infection #1 and #2.** **A** Experiment #1. **B** Experiment #2. Asterisk the mortality rate at the end of the experiment was significantly different from the negative control (*p* < 0.01 by Fisher’s exact test).

The cumulative mortalities in rainbow trout injected with VHSV DK-3592B (GIa), NO-2007-50-385 and 4p168 were 65, 20 and 0%, respectively (Figure 1A). The cumulative mortality observed in rainbow trout immersed with DK-3592B for 1 h was 75%. Rainbow trout bath challenged with NO-2007-50-385 for 1 and 6 h, resulted in cumulative mortalities of 15 and 20%, respectively. No mortality was observed in the rainbow trout immersed with 4p168 for 1 and 6 h. No mortality was observed in the negative control group (Figure 1A; Table 2). The mortalities in the groups infected with DK-3592B by i.p. injection (*p* < 0.0001) and 1 h immersion (*p* < 0.0001) were significantly higher than the negative control group.

**Table 2:** VHSV detection by reverse-transcription PCR and virus re-isolation in cell cultures inoculated with organs from fish that died during the experimental observation period, or those that survived and were harvested at 46 dpe for experiment 1, and 29 dpe for experiment 2.

Experiment	Group (genotype)	Treatment (infectious route/negative control)	Cumulative mortality (%)	Dead fish (no. of positive fish/no. of tested fish)				Surviving fish (no. of positive fish/no. of tested fish)			
				Kidney + spleen		Brain		Kidney + spleen		Brain	
				Virus re-isolation	RT-PCR	Virus re-isolation	RT-PCR	Virus re-isolation	RT-PCR	Virus re-isolation	RT-PCR
1	DK-3592B (Ia)	IP	65	12/13	11/13	13/13	9/13	0/7	0/7	0/7	0/7
		Immersion 1 h	75	15/15	14/15	15/15	15/15	0/5	0/5	0/5	0/5
	NO-2007-50-385 (III)	IP	20	3/4	3/4	4/4	4/4	0/16	0/16	0/16	0/16
		Immersion 1 h	15	2/3	2/3	3/3	3/3	1/17	0/17	2/17	1/17
		Immersion 6 h	20	3/4	3/4	4/4	4/4	0/16	0/16	1/16	0/16
	4p168 (III)	IP	0	–	–	–	–	0/20	0/20	0/20	0/20
		Immersion 1 h	0	–	–	–	–	0/20	0/20	0/20	0/20
		Immersion 6 h	0	–	–	–	–	0/20	0/20	0/20	0/20
	Negative control	IP	0	–	–	–	–	0/20	0/20	0/20	0/20
		Immersion 1 h	0	–	–	–	–	0/20	0/20	0/20	0/20
2	DK-3592B (Ia)	Immersion 1 h	65	13/13	12/13	13/13	11/13	0/7	0/7	1/7	1/7
	NO-2007-50-385 (III)	Immersion 1 h	5	1/1	1/1	1/1	1/1	2/19	0/19	4/19	3/19
		Immersion 6 h	35	7/7	7/7	7/7	7/7	3/13	0/13	3/13	3/13
	4p168 (III)	Immersion 1 h	0	–	–	–	–	0/20	0/20	0/20	0/20
		Immersion 6 h	0	–	–	–	–	0/20	0/20	0/20	0/20
	Negative control	Immersion 6 h	0	–	–	–	–	0/20	0/20	0/20	0/20

VHSV was re-isolated from all the brain and most of the kidney/spleen samples collected from rainbow trout that died due to infection with DK-3592B and NO-2007-50-385 (Table 2). From the fish that survived infection by immersion for 1 and 6 h with NO-2007-50-385, VHSV was re-isolated from 3 out of 33 (3/33) brain samples and in 1/33 kidney/spleen samples. No VHSV was isolated from any of the fish that survived exposure to DK-3592B or 4p168, or from any of the non-exposed control fish.

VHSV was detected by RT-PCR from most brain and kidney/spleen samples collected from rainbow trout dying due to infection with DK-3592B and NO-2007-50-385 (Table 2). No VHSV was detected by RT-PCR in any of the samples from fish that survived VHSV infections except for 1/17 brain samples from the fish surviving 1 h immersion with NO-2007-50-385 (Table 2).

### Infection experiment 2

In infection trial #2, a cumulative mortality of 65% was observed in rainbow trout immersed with DK-3592B for 1 h (Table 2; Figure 1B). Cumulative mortalities of 5 and 35% were observed in rainbow trout bath challenged with NO-2007-50-385 for 1 and 6 h, respectively. No mortality was observed in rainbow trout immersed with 4p168 for 1 and 6 h, or in the negative control group. The mortalities in the group infected with DK-3592B (*p* < 0.0001) and NO-2007-50-385 by immersion for 6 h (*p* < 0.005) were significantly higher than the negative control group. In addition, the mortalities in the group infected by 1 h immersion with NO-2007-50-385 were significantly lower than the group infected with DK-3592B (*p* < 0.0001) and 6 h immersion with NO-2007-50-385 (*p* < 0.05).

VHSV was re-isolated from all the kidney/spleen and brain samples from fish that died due to infection with DK-3592B and NO-2007-50-385 (Table 2). In addition VHSV was re-isolated from 5/32 kidney/spleen and 7/32 brain samples from fish surviving infection with NO-2007-50-385. The frequency of re-isolation of virus from brain samples was slightly higher than from the kidney/spleen samples. VHSV was not isolated from any of the fish infected with 4p168 or from the non-infected control fish.

VHSV was detected by RT-PCR in all of the brain and most of the kidney/spleen samples from fish that died due to DK-3592B and NO-2007-50-385 infection. Viral RNA was not detected by RT-PCR in any of the samples from surviving fish infected with 4p168 or from any of the non-infected control fish, but was detected in 6/32 brain samples from fish infected with NO-2007-50-385 (Table 2).

### Sequential titration of VHSV from fish sampled in experiment 2

#### Examination of sacrificed fish

The sequential viral titres in gill, brain, heart and kidney from surviving fish after infection by immersion with DK-3592B and NO-2007-50-85 are shown in Table 3. No virus was detected in the fish infected with 4p168 isolate at any time sampled, and therefore those results are not shown in the Table 3. VHSV could be detected from the gills (10^3.5^ TCID_50_/g), heart (10^3.6^ TCID_50_/g) and kidney (<10^1.3^ TCID _50_/g) in 1 of the 3 DK-3592B infected fish sacrificed 1 dpe. Virus under the limit of titration was also detected in gills from another fish 1 dpe to DK-3592B. No virus was detected in any fish 1 day after infection with the other two viruses. At 3 dpe, virus was detected in gills from 1 fish, brain and heart from 2 fish, and kidney from all the 3 fish sacrificed in the DK-3592B group. In the 6 h immersion NO-2007-50-385 group, virus was detected in the brain, heart and kidney from 2 of 3 fish. At five dpe to DK-3592B, VHSV was detected in most of the organs from the three sacrificed fish, except from the brain of 1 fish. The titres, ranging from 10^3.9^ to 10^9.4^ TCID_50_/g, were in most cases higher than in samples collected earlier. In samples collected 5 dpe from 3 NO-2007-50-385 1 h immersion infected fish, virus was detected in all the organs from 1 of 3 fish, and was detected in the gill, brain and kidney from another fish. In the fish immersed 6 h with NO-2007-50-385, virus was detected in all tested organs from 1 of 3 fish, and was detected in the gill and heart from another fish. The virus was detected in the 4 organs from the 3 fish sacrificed 7 dpe with DK-3592B, except in the brain of 2 fish. In the fish sacrificed 7 dpe in the NO-2007-50-385 1 h group, VHSV was detected in kidney with a titre of 10^4.1^ TCID_50_/g, and in gill from one fish with a titre under the limit of titration. In the case of the 6 h immersion NO-2007-50-385 fish, virus titre was detected in all the organs tested from 1 fish. Virus was detected 13 dpe in all organs tested from the two sacrificed fish infected with DK-3592B. At this point no virus was detected in the three fish collected from the group infected with NO-2007-50-385 for 1 h, and was detected in heart from 1 fish and in all organs from another of the three fish examined 13 dpe for 6 h with NO-2007-50-385. On 20 dpe, VHSV was only detected in the brain from 2 of the 3 sacrificed fish in the NO-2007-50-385 1 h immersion group, whereas all other samples were negative.

**Table 3:** Sequential titration of VHSV in the gills, brain, and kidney of fish sampled at pre-determined time points in experiment 2, after exposure by immersion to various VHSV isolates.

Days post exposure	Group	Individual no.	TCID_50_/g organ				
			Gill	Brain	Heart	Kidney	
1	DK-3592B	1	10^3.5^	0^a^	10^3.6^	<10^1.3b^	
		2	0	0	0	0	
		3	<10^1.3^	0	0	0	
	NO-2007-50-385 1 h	1	0	0	0	0	
		2	0	0	0	0	
		3	0	0	0	0	
	NO-2007-50-385 6 h	1	0	0	0	0	
		2	0	0	0	0	
		3	0	0	0	0	
3	DK-3592B	1	0	0	0	<10^1.3^	
		2	0	<10^1.3^	10^4.2^	10^5.3^	
		3	10^3.9^	<10^1.3^	10^4.9^	10^6.0^	
	NO-2007-50-385 1 h	1	0	0	0	0	
		2	0	0	0	0	
		3	0	0	0	0	
	NO-2007-50-385 6 h	1	0	0	0	0	
		2	0	<10^1.3^	10^3.6^	10^7.2^	
		3	0	10^4.7^	10^5.9^	10^7.1^	
5	DK-3592B	1	10^8.1^	10^6.8^	10^9.4^	10^9.3^	
		2	10^6.9^	10^5.9^	10^8.2^	10^7.5^	
		3	10^3.9^	0	10^5.1^	10^3.9^	
	NO-2007-50-385 1 h	1	10^6.9^	10^5.1^	10^8.1^	10^7.6^	
		2	10^4.0^	10^3.7^	0	<10^1.3^	
		3	0	0	0	<10^1.3^	
	NO-2007-50-385 6 h	1	10^5.9^	10^7.3^	10^7.2^	10^7.7^	
		2	10^3.2^	0	10^5.2^	0	
		3	0	<10^1.3^	0	0	
7	DK-3592B	1	10^5.9^	10 ^4.9^	10^8.2^	10^6.2^	
		2	10^4.1^	0	10^6.8^	10^4.5^	
		3	10^3.6^	0	10^6.3^	10^3.6^	
	NO-2007-50-385 1 h	1	0	0	0	0	
		2	0	0	0	0	
		3	<10^1.3^	0	0	10^4.1^	
	NO-2007-50-385 6 h	1	<10^1.3^	0	<10^1.3^	0	
		2	10^4.9^	10 ^6.0^	10^7.0^	10^6.5^	
		3	0	0	0	0	
13	DK-3592B	1	10^8.3^	<10^1.3^	10^8.2^	10^4.0^	
		2	10^5.7^	10^4.7^	10^7.6^	10^6.0^	
	NO-2007-50-385 1 h	1	0	0	0	0	
		2	0	0	0	0	
		3	0	0	0	0	
	NO-2007-50-385 6 h	1	0	0	<10^1.3^	0	
		2	10^4.5^	<10^1.3^	10^5.1^	10^5.1^	
		3	0	0	0	0	
20	NO-2007-50-385 1 h	1	0	0	0	0	
		2	0	10^3.1^	0	0	
		3	0	10^3.6^	0	0	
	NO-2007-50-385 6h	1	0	0	0	0	
		2	0	0	0	0	

^a^0, Not detected

^b^<10^1.3^, Positive but under limit for titration (<10^1.3^ TCID _50_/g)

#### Examination of mortalities in infection trial 2

The viral titres in gill, brain, heart and kidney from fish that died due to infection with the VHSV isolates are shown in Table 4. First mortalities were observed 6, 7 and 12 dpe to DK-3592B, NO-2007-50-385 1 h and NO-2007-50-385 6 h, respectively. VHSV was detected in the 4 tested organs from all examined fish. The median titres were 10^6.4^, 10^5.6^, 10^8.0^ and 10^6.8^ TCID_50_/g in gill, brain, heart and kidney, respectively. The heart showed the highest titres in 9 of 11 fish, and brain the lowest in 10 of 11 fish.

**Table 4:** Titration of VHSV in the gill, brain, heart and kidney of fish that died during experiment 2.

Group	Days post exposure	Individual no.	TCID_50_/g organ			
			Gill	Brain	Heart	Kidney
DK-3592B	6	1	10^7.3^	10^5.7^	10^8.8^	10^7.2^
	7	1	10^5.8^	10^5.0^	10^7.6^	10^6.9^
	8	1	10^5.8^	10^4.8^	10^7.3^	10^4.7^
	10	1	10^4.4^	10^4.1^	10^8.0^	10^6.4^
	11	1	10^4.4^	10^3.8^	10^8.3^	10^6.5^
	12	1	10^5.9^	10^3.8^	10^7.7^	10^6.0^
NO-2007-50-385 1 h	7	1	10^6.6^	10^6.5^	10^6.6^	10^7.6^
	18	1	10^4.8^	10^3.7^	10^5.5^	10^4.5^
NO-2007-50-385 6 h	12	1	10^5.9^	10^4.6^	10^7.9^	10^4.9^
	13	1	10^5.4^	10^4.7^	10^6.0^	10^6.2^
	19	1	10^5.6^	10^4.3^	10^8.0^	10^6.0^
Median titres			10^6.4^	10^5.6^	10^8.0^	10^6.8^

### Multiplication of VHSV genotype III in fish cell lines

Multiplication of the isolates DK-3592B, NO-2007-50-385 and 4p168 in three different cell lines is shown in Table 5. No remarkable differences were observed between the three VHSV isolates in the BF-2 and EPC cell lines. But the titre of the non-virulent VHSV isolate 4p168 in the rainbow trout cell line RTG-2 was 3–4 logs lower than the two virulent VHSV isolates, and it was also lower than the 4p168 titres attained in BF-2 and EPC cells.

**Table 5:** Titration of VHSV isolates in 3 cell lines.

Isolates (genotype)	Titre (TCID_50_/mL)			
	BF-2	EPC	RTG-2	
	Sample A and B	Sample A and B	Sample A and B	
DK-3592B (GIa)	10^8.8^, 10^9.3^	10^9.3^, 10^9.0^	10^8.3^, 10^8.3^	
NO-2007-50-385 (GIII)	10^7.8^, 10^7.8^	10^8.0^, 10^7.8^	10^7.3^, 10^7.3^	
4p168 (GIII)	10^8.6^, 10^8.0^	10^8.0^, 10^7.8^	10^3.8^, 10^4.3^	

### Comparative analysis of amino acid (aa) alignments

**Figure 2:**
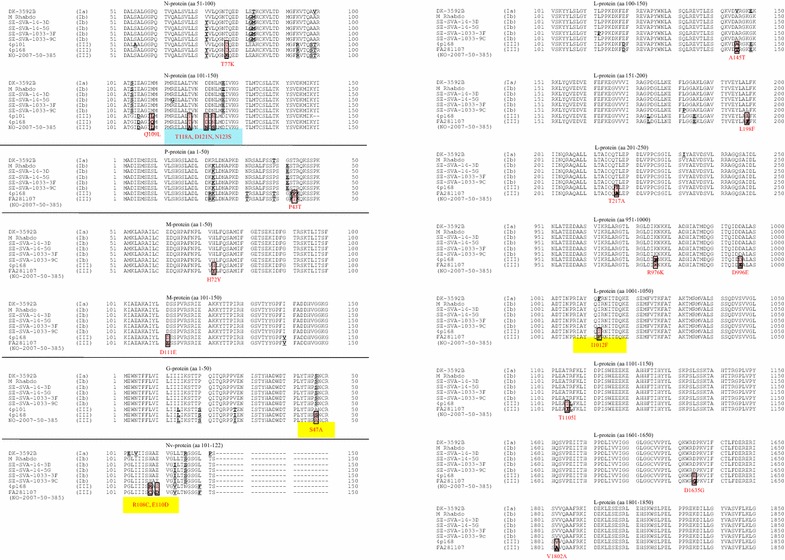
**Partial amino acid alignments of viral proteins from various VHSV isolates.** The part shaded red are amino acid substitutions between 4p168 as a representative for a non-virulent VHSV genotype III isolate and NO-2007-50-385 as a representative for a virulent VHSV genotype III isolate to rainbow trout. The part shaded blue (aa 118–123 of the N-protein) and the part shaded yellow (aa 47 of the G-protein, aa 108 and 110 of the Nv-protein, and aa 1012 of the L-protein) are referred to in the “Discussion”.

For comparative analysis of aa substitution sites between rainbow trout non-virulent and virulent VHSV genotype III isolates, the whole genome sequence of the low virulent isolate 4p168 was sequenced and submitted to DDBJ as accession no. AB672616. The entire aa sequence of 4p168 was aligned with the aa sequence of the virulent GIII isolate. Since FA281107 [19] is synonym to NO-2007-50-385, the N- (AB675945) and G-protein (EU547740) of NO-2007-50-385 and the P, M, Nv and L-gene/protein of FA281107 (EU481506) were used to represent a virulent GIII isolate for comparison with those of the non-virulent 4p168 isolate. As a result, 5 aa substitutions in the N-protein, 1 in the phospho (P)-protein, 2 in the matrix (M)-protein, 1 in the G-protein, 2 in the Nv-protein and 9 substitutions in the L-protein were detected (Table 6; Figure 2; Additional file 2).

**Table 6:** Comparative analysis of amino acid.

Protein	Product size (aa)	Substitution (n)	Identity (%)	Substituted (aa)	Substitution position (AA)
N	404	5	98.8	T-K, Q-L, T-A, D-N, N-S	77, 109, 118, 121, 123
P	222	1	99.6	P-T	43
M	201	2	99.0	H-Y, D-E	72, 111
G	507	1	99.8	S-A	47
Nv	122	2	98.4	R-C, E-D	108, 110
L	1984	9	99.5	A-T, L-F, T-A, R-K, D-E,	145, 198, 217, 976, 996,
				I-F, T-I, D-G, V-A	1012, 1105, 1635, 1805

Amino acid (aa) substitution among the VHSV GIII isolates with different virulence properties in rainbow trout (4p168 vs NO-2007-50-385 or FA281107)

## Discussion

This paper is the first report of a direct comparison between 2 VHSV Genotype III isolates of different virulence to rainbow trout. The studies included comparative infection trials, cell culture susceptibility tests and analysis of the amino acid sequences of the entire viral genome. In fact, the complete genome sequencing of the non-virulent 4p168 isolate must be considered an important achievement in this study. We have demonstrated that the length of the exposure to the virus by immersion is important for the level of mortality in the infection trials. In addition, we have suggested that only 3 aa substitutions in the N-protein are enough for modulating the virulence of VHSV GIII isolates in rainbow trout.

Dale et al. [18] reported that the isolate NO-2007-50-385 was highly virulent for rainbow trout by i.p. injection and immersion. However, this study did not include comparisons with other VHSV GIII isolates known to be low or non-virulent to rainbow trout [8]. However the two infection trials of the present study have corroborated those findings by demonstrating moderate virulence of NO-2007-50-385 and non-virulence of 4p168 to rainbow trout. Moreover, it was shown that the mortality induced by NO-2007-50-385 depended on the time of exposure by immersion, clearly in experiment #2. By conducting sequential titrations of a number of organs from fish sacrificed during trial #2, it was shown that virus titres in fish exposed for 6 h raised more quickly and persisted for longer than in fish exposed for 1 h. These results indicate that the time of exposure to VHSV has a significant effect on the virulence to the fish. The low mortality and the low virus re-isolation rate in the group exposed 1 h to NO-2007-50-385 may suggest that the innate immunity mechanisms of the fish were more successful at controlling the quantity of infectious virus that gained entry during the shorter exposure. In general, although the viral concentration in aquaculture is lower than under experimental infection, the fish will be exposed to virus for a long time. Therefore, when the virulence of a virus isolate is studied, the time of exposure seem to play a significant role and should be taken into consideration.

The highest titres were observed in heart, indicating that it is important to collect this organ when examining fish for VHSV. On the other hand it appears that the virus in later stages will hide in the brains of a small number of surviving fish exposed to NO-2007-50-385 (Table 2). In a previous study on the virulence of VHSV genotype IVb isolates to a number of different fish species, VHSV was detected in the brain from some of the surviving fish, and not detected in pooled samples of kidney/spleen [32]. Iida et al. [33] reported that VHSV GIVa was isolated only from the brain and heart of Japanese flounder 6 weeks post immersion challenge. This study also indicates that VHSV is retained in the brain when compared to kidney and spleen in late stages of the infections.

Compared with RT-PCR a few more samples were positive by virus isolation. The discrepancy may be caused by differences in the quantity of sample included in each method. A recent validation of a new real time RT-PCR [34] also showed lower sensitivity of this conventional RT-PCR method in comparison to virus isolation and real time RT-PCR assay.

Previous studies on mammal rhabdoviruses, have successfully identified virulence determinants of rabies virus, whereby a few point mutations of aa within the G-protein was sufficient to have a dramatic impact on the virulence [35, 36]. These studies demonstrate the significant impact the G-protein in effecting virulence, it also emphasizes the notion that only a few aa substitutions are necessary to determine virulence amongst rhabdoviruses for a prescribed host.

With regard to VHSV virulence determinants for rainbow trout, Béarzotti et al. [9] and Stone et al. [15] reported that the G-protein seems to play an important role in the virulence of VHSV to rainbow trout. The G-protein is also responsible for development of neutralizing antibodies and is the only viral protein that can induce protective immunity in fish [12, 37]. Neutralization of VHSV infectivity to cell cultures and fish by monoclonal antibodies against the viral G-protein has been demonstrated [12, 14, 37]. Therefore, it was assumed that determinants for virulence in rainbow trout should be found in this gene, and several reports have been focused on comparative studies of the G-protein in high- and non-virulent isolates [10, 11]. However, no clear results have been provided yet pointing at which substitutions of nucleotide and/or amino acid in the G-gene/protein of VHSV are a key for triggering pathogenicity in fish. Recent reports [16, 17] suggest that any non G-gene/protein may have a role as virulence determinants to rainbow trout as well.

From the comparative analysis of all protein coding regions from rainbow trout non-virulent and virulent VHSV genotype III isolates, only one aa substitution in the G-protein was demonstrated and at substitution, at position aa 47, does not seem to be related with virulence in rainbow trout, since the aa in this position of both, the high virulent isolate DK-3592B and the non-virulent isolate 4p168, is serine. Determinants of virulence in rainbow trout should thus be found elsewhere than in the viral G-protein.

Based on the reaction patterns with mAb VHS-3.75 and aa alignments of the full genome sequences of VHSV GIII, we suggest that substitutions of aa in the N-protein region at aa position 118–123 of the virulent NO-2007-385-50 (AVNNDS) and the non-virulent 4p168 (TVNDDN) isolates could be related to virulence of VHSV GIII in rainbow trout. That region is the epitopic part of mAb VHS-3.75 which shows a different reaction pattern between the NO-2007-50-385 and 4p168 isolates [22]. Particularly, three aa substitutions at 118 (threonine → alanine), 121 (aspartic acid → asparagine) and 123 (asparagine → serine) of the N-protein might be the site for causing changes in virulence from low to high for rainbow trout. Since the NDS domain (position 121–123 of virulent isolates) is well known as a N-linked glycosylation site, molecular conformation of the region of those isolates could be very different.

Using reverse genetics, Kim et al. [16] reported that a point mutation from I (isoleucine) to F (phenylalanine) at position 1092 in the L-protein of GIII and GIVa VHSV isolates can increase the virulence in an in vitro trial. Although the aa in position 1012 of NO-2007-50-385 and DK-3592B is F, it is I in the GIb isolates SE-SVA-14-3D and SE-SVA-1033-3F of medium/high (30–90% mortalities) virulence to rainbow trout [38, 39]. This means that although being a candidate, the aa substitution in position 1012 of the L-protein clearly is not the only determinant for virulence in rainbow trout.

Judging from the results of the aa alignments, one possibility could be that the Nv-protein participated in virulence. Two aa substitutions in the Nv-protein were found in the comparative study between 4p168 and NO-2007-50-385. One of those, in position 110, is shared between 4p168 and DK-3592B isolates, and can therefore not be a singular virulence determinant when applying the same logic than above. The other aa substitution in position 108 is more interesting, as both 4p168 and NO-2007-50-385 isolates differ from each other and from all other isolates, including DK-3592B. A few reports indicate that the Nv-genome of VHSV plays an important role in the virulence of GIVa and GIVb [40, 41]. However, Einer-Jensen et al. [17] reported that differences in virulence among phylogenetically distinct isolates of VHSV are not explained by variability of the Nv. Therefore, regarding the virulence to rainbow trout, future studies on aa substitutions in Nv are needed to determine its role in the virulence of VHSV to that fish species.

One at position 43 in the P-protein and 2 aa substitutions at position 72 and 111 in the M-protein are found by comparison of aa alignments. The role of these substitutions in the virulence of VHSV might also need future studies.

The DK-3592B and NO-2007-50-385 isolates showed high titre in RTG-2 cells, but the titre of 4p168 was low in that cell. This result suggests that certain processes in viral replication in rainbow trout cells are different between NO-2007-50-385 and 4p168.

It may be necessary for VHSV to pass a number of barriers before it becomes virulent in rainbow trout. The first barrier for the virus is its entry into the host cells. The second barrier is its replication in the cells. GIa isolates such as DK-3592B can easily pass through the first barrier, since the G-protein may have high affinity to cells of rainbow trout [42]. Continuously, it can easily pass to a second barrier, since it also can propagate in the rainbow trout cells and, as a result, the infected fish dies. Since there is only one aa substitution of the G-protein between NO-2007-50-385 and 4p168 isolates, it is supposed that the intensity of infection of the host cells must be similar. Nevertheless, as for the normal genotype III viruses such as the 4p168 isolate, the fish does not die because it cannot propagate with the same efficiency in rainbow trout. However, NO-2007-50-385 can propagate in the host cell and thereby kill the fish.

The isolation of NO-2007-50-385 might be a result of VHSV GIII being present as virus populations composed of a cloud of variants in the wild and farm environment. Other reports revealed that VHSV GIb isolates might become virulent in farmed rainbow trout in marine environment [43, 44]. To this regard, marine isolates of VHSV may be a potential threat to the farming industry if provided with the opportunity to adapt under intensive farming conditions.

## Additional files

10.1186/s13567-015-0303-z **Primers used for complete genomic sequencing of VHSV 4p168.**

10.1186/s13567-015-0303-z **Partial amino acid alignments of viral proteins from various VHSV isolates.** The part shaded red are amino acid substitutions between 4p168 as a representative for a non-virulent VHSV genotype III isolate and NO-2007-50-385 as a representative for a virulent VHSV genotype III isolate to rainbow trout. The part shaded blue (aa 118-123 of the N-protein) and the part shaded yellow (aa 47 of the G-protein, aa 108 and 110 of the Nv-protein, and aa 1012 of the L-protein) are referred to in the “Discussion”.
