# Supplementary material for: Virulence of viral haemorrhagic septicaemia virus (VHSV) genotype III in rainbow trout
Source: Vet Res. 2016 Jan 8;47:4. doi: 10.1186/s13567-015-0303-z (PMC4705761; doi:10.1186/s13567-015-0303-z)
Supplement: Supplementary file 1 — 10.1186/s13567-015-0303-z Primers used for complete genomic sequencing of VHSV 4p168. [file 13567_2015_303_MOESM1_ESM.docx]

**Primer Sequence**

VH common FRONT term2 new CDTAAGTRGCAAAAAGTTTTCAAGTTTG

VN N mid F1 TYTTGTCCACMGAGTACTTG

VH N mid F2 GTGGACAARATGATCAAGTAC

VH N mid R1 CAAGTACTCKGTGGACAARA

VH N mid R2 GTACTTGATCATYTTGTCCAC

VH N rear F GCCCGCAACTTCAGGAGC

VH N REV 216 TCAACCTCACCAGGTACAAGCAC

VH N REV NEST170 CGATCCTGATGTCATTCAAAC

VH P front R CYTTRGGGGCGTTGTCTAG

VH P mid F GGAAGAAGACCGACAACATAC

VH P mid R AGTATGTTGTCGGTCTTCTTC

VH P rear F CATYGCCATGAAGAAGTTCAAG

VH M front R ATGGTKGAGACACGGTCCTC

VH M midrear F AGACATGGGAGTGTGACTTA

VH M midrear R TAAGTCACACTCCCATGTCT

VH G front R TTCATCCARATGCAGGARGGTTC

VH G front F GAACCYTCCTGCATYTGGATGAA

VH G midrear F TTTCTCCTMTCAAAGTTTCGTCC

VH G midrear R GGACGAAACTTTGAKAGGAGAAA

VH G rear F TCTCCAACACATCYGATCTYTC

VH G rear R GARAGATCRGATGTGTTGGAGA

VH NV front F GACCCAAGYAACTACCTCAAC

VH NV front R GTTGAGGTAGTTRCTTGGGTC

VH LfrontF GTACCAGCTGGTGCACCTCAG

VH LfrontR ATCCTAACTCATTGCTCTGTGTC

VH LfrontF-2 ATTTAAAGAGGGGGTGGTGG

VH LfrontR-2 GAGTCCAGGATCTGTCAGCC

VH L2R-2 GATGATTTTGAAGAGGGCCA

VH L2F GCTCTCTTCCAATATTTTGGACTG

VH L2R ATGAGTCGCTTGGTTTGTCACTG

VH L2F-2 TGGCCACTTAGTGACACCCT

VH L5R-2 TTTCTGTGGTGTCTCTTCCTGA

VH L3R GACTTTTACTGATGTGGATGTATC

VH L2F-3 AACGCCAGTTCAACTCCTTG

VH L5R-3 ATCCGTCCACAATCCATGTT

VH L2F-4 TGAGGAGTGGCCTGACTCTT

VH L5R-4 AGCAAGGGTAAGGTGCTGTG

VH L5R-5 TGAGTAACGTAGTCTCCCACACC

VH L4F CGAAAACCATGCGAATGGTTGC

VH L4F-2 GGGAGACCATGATTAAGGCA

VH 5RACES1-2 CTTGTGCTTGAAGTGAGGGG

VH L5R CGTTTCCTTTGGGATGTCCAAG

VH 5RACES1-3 AGGACCAAGGTCACAGGATG

VH 5RACES1-4 TCTTCCCAGCTTTCTTGTCC

VH L8.5comfF GTCAAGACCAAATCTCTGTTC

VH 5RACES1-5 AACAGAGGTGAGGTTGCACA

VH 5RACERT5P GCTCATCACTCTTCTTGAAGAAG

VH 5RACES1 TTCTCGGTGATGGCATCCGAG

VH 5endR GGCGATTCCATCACTGTTCT
