# Supplementary material for: Virulence of viral haemorrhagic septicaemia virus (VHSV) genotype III in rainbow trout
Source: Vet Res. 2016 Jan 8;47:4. doi: 10.1186/s13567-015-0303-z (PMC4705761; doi:10.1186/s13567-015-0303-z)
Supplement: Supplementary file 2 — 10.1186/s13567-015-0303-z Partial amino acid alignments of viral proteins from various VHSV isolates. The part shaded red are amino acid substitutions between 4p168 as a representative for a non-virulent VHSV genotype III isolate and NO-2007-50-385 as a representative for a virulent VHSV genotype III isolate to rainbow trout. The part shaded blue (aa 118-123 of the N-protein) and the part shaded yellow (aa 47 of the G-protein, aa 108 and 110 of the Nv-protein, and aa 1012 of the L-protein) are referred to in the “Discussion”. [file 13567_2015_303_MOESM2_ESM.pptx]

## Slide 1
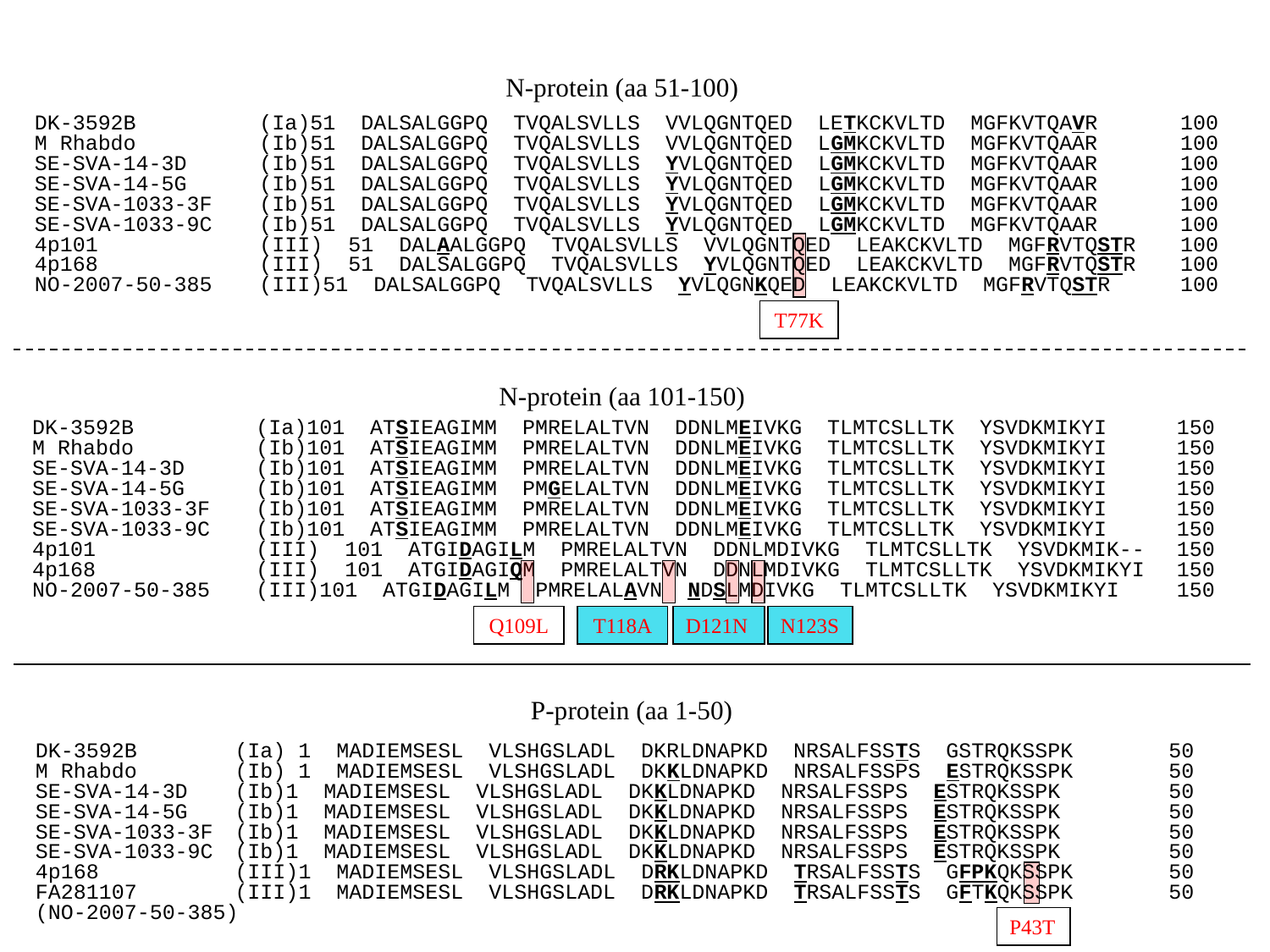

N-protein (aa 51-100)
DK-3592B	(Ia)	51 DALSALGGPQ TVQALSVLLS VVLQGNTQED LETKCKVLTD MGFKVTQAVR 	100
M Rhabdo	(Ib)	51 DALSALGGPQ TVQALSVLLS VVLQGNTQED LGMKCKVLTD MGFKVTQAAR 	100
SE-SVA-14-3D	(Ib)	51 DALSALGGPQ TVQALSVLLS YVLQGNTQED LGMKCKVLTD MGFKVTQAAR	100
SE-SVA-14-5G	(Ib)	51 DALSALGGPQ TVQALSVLLS YVLQGNTQED LGMKCKVLTD MGFKVTQAAR	100
SE-SVA-1033-3F	(Ib)	51 DALSALGGPQ TVQALSVLLS YVLQGNTQED LGMKCKVLTD MGFKVTQAAR	100
SE-SVA-1033-9C	(Ib)	51 DALSALGGPQ TVQALSVLLS YVLQGNTQED LGMKCKVLTD MGFKVTQAAR	100
4p101	(III) 	 51 DALAALGGPQ TVQALSVLLS VVLQGNTQED LEAKCKVLTD MGFRVTQSTR 	100
4p168	(III) 	 51 DALSALGGPQ TVQALSVLLS YVLQGNTQED LEAKCKVLTD MGFRVTQSTR	100
NO-2007-50-385	(III)	51 DALSALGGPQ TVQALSVLLS YVLQGNKQED LEAKCKVLTD MGFRVTQSTR	100
T77K
N-protein (aa 101-150)
DK-3592B	(Ia)	101 ATSIEAGIMM PMRELALTVN DDNLMEIVKG TLMTCSLLTK YSVDKMIKYI	150
M Rhabdo	(Ib)	101 ATSIEAGIMM PMRELALTVN DDNLMEIVKG TLMTCSLLTK YSVDKMIKYI	150
SE-SVA-14-3D	(Ib)	101 ATSIEAGIMM PMRELALTVN DDNLMEIVKG TLMTCSLLTK YSVDKMIKYI	150
SE-SVA-14-5G	(Ib)	101 ATSIEAGIMM PMGELALTVN DDNLMEIVKG TLMTCSLLTK YSVDKMIKYI	150
SE-SVA-1033-3F	(Ib)	101 ATSIEAGIMM PMRELALTVN DDNLMEIVKG TLMTCSLLTK YSVDKMIKYI	150
SE-SVA-1033-9C	(Ib)	101 ATSIEAGIMM PMRELALTVN DDNLMEIVKG TLMTCSLLTK YSVDKMIKYI	150
4p101	(III) 	 101 ATGIDAGILM PMRELALTVN DDNLMDIVKG TLMTCSLLTK YSVDKMIK--	150
4p168	(III) 	 101 ATGIDAGIQM PMRELALTVN DDNLMDIVKG TLMTCSLLTK YSVDKMIKYI	150
NO-2007-50-385 	(III)	101 ATGIDAGILM PMRELALAVN NDSLMDIVKG TLMTCSLLTK YSVDKMIKYI	150
Q109L
T118A
D121N
N123S
P-protein (aa 1-50)
DK-3592B	(Ia)	 1 MADIEMSESL VLSHGSLADL DKRLDNAPKD NRSALFSSTS GSTRQKSSPK	50
M Rhabdo	(Ib)	 1 MADIEMSESL VLSHGSLADL DKKLDNAPKD NRSALFSSPS ESTRQKSSPK	50
SE-SVA-14-3D	(Ib)	1 MADIEMSESL VLSHGSLADL DKKLDNAPKD NRSALFSSPS ESTRQKSSPK	50
SE-SVA-14-5G	(Ib)	1 MADIEMSESL VLSHGSLADL DKKLDNAPKD NRSALFSSPS ESTRQKSSPK	50
SE-SVA-1033-3F	(Ib)	1 MADIEMSESL VLSHGSLADL DKKLDNAPKD NRSALFSSPS ESTRQKSSPK	50
SE-SVA-1033-9C	(Ib)	1 MADIEMSESL VLSHGSLADL DKKLDNAPKD NRSALFSSPS ESTRQKSSPK	50
4p168	(III)	1 MADIEMSESL VLSHGSLADL DRKLDNAPKD TRSALFSSTS GFPKQKSSPK	50
FA281107	(III)	1 MADIEMSESL VLSHGSLADL DRKLDNAPKD TRSALFSSTS GFTKQKSSPK	50
(NO-2007-50-385)
P43T

## Slide 2
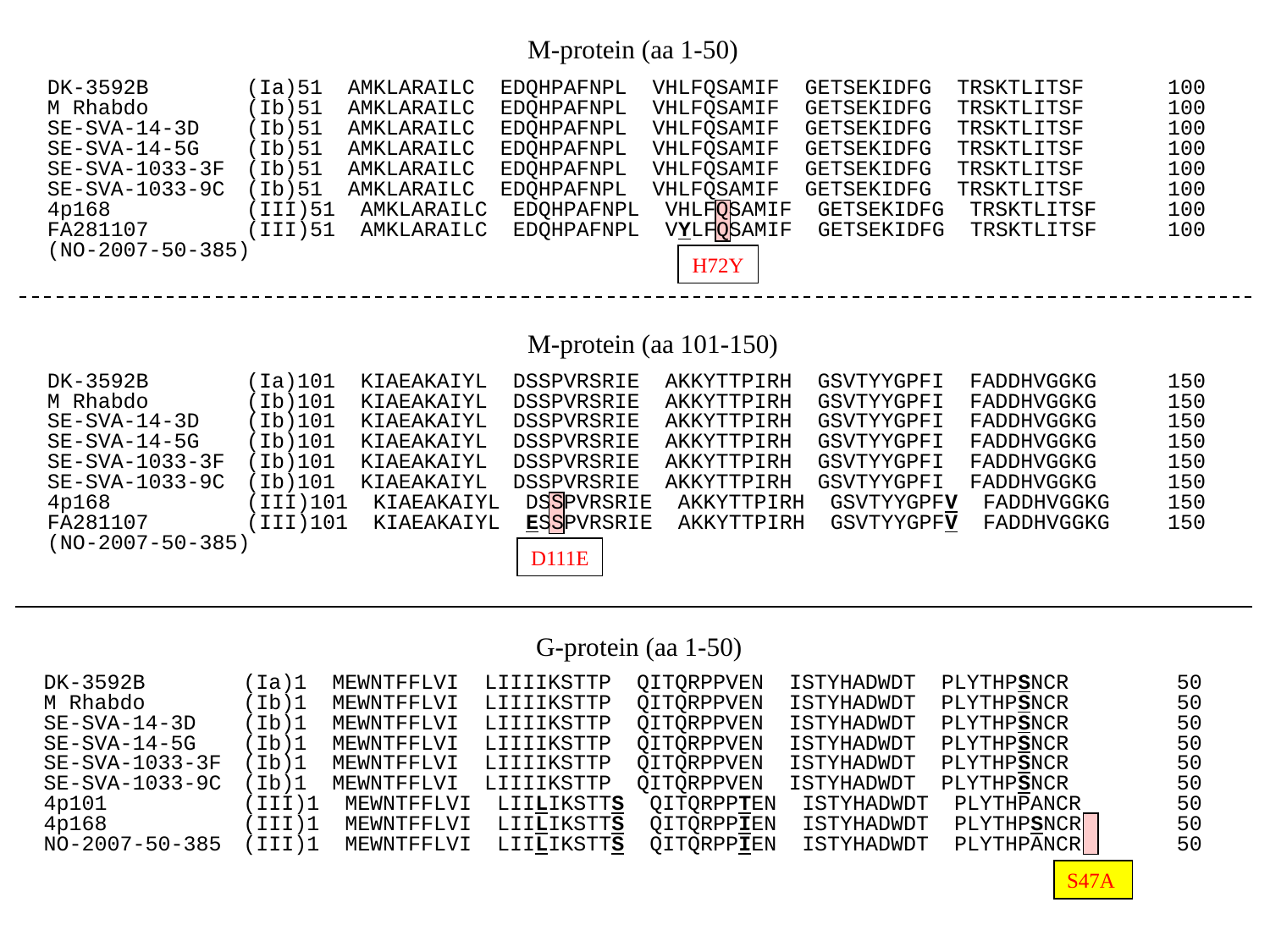

M-protein (aa 1-50)
DK-3592B	(Ia)	51 AMKLARAILC EDQHPAFNPL VHLFQSAMIF GETSEKIDFG TRSKTLITSF	100
M Rhabdo	(Ib)	51 AMKLARAILC EDQHPAFNPL VHLFQSAMIF GETSEKIDFG TRSKTLITSF	100
SE-SVA-14-3D	(Ib)	51 AMKLARAILC EDQHPAFNPL VHLFQSAMIF GETSEKIDFG TRSKTLITSF	100
SE-SVA-14-5G	(Ib)	51 AMKLARAILC EDQHPAFNPL VHLFQSAMIF GETSEKIDFG TRSKTLITSF	100
SE-SVA-1033-3F	(Ib)	51 AMKLARAILC EDQHPAFNPL VHLFQSAMIF GETSEKIDFG TRSKTLITSF	100
SE-SVA-1033-9C	(Ib)	51 AMKLARAILC EDQHPAFNPL VHLFQSAMIF GETSEKIDFG TRSKTLITSF	100
4p168	(III)	51 AMKLARAILC EDQHPAFNPL VHLFQSAMIF GETSEKIDFG TRSKTLITSF	100
FA281107	(III)	51 AMKLARAILC EDQHPAFNPL VYLFQSAMIF GETSEKIDFG TRSKTLITSF	100
(NO-2007-50-385)
H72Y
M-protein (aa 101-150)
DK-3592B	(Ia)	101 KIAEAKAIYL DSSPVRSRIE AKKYTTPIRH GSVTYYGPFI FADDHVGGKG	150
M Rhabdo	(Ib)	101 KIAEAKAIYL DSSPVRSRIE AKKYTTPIRH GSVTYYGPFI FADDHVGGKG	150
SE-SVA-14-3D	(Ib)	101 KIAEAKAIYL DSSPVRSRIE AKKYTTPIRH GSVTYYGPFI FADDHVGGKG	150
SE-SVA-14-5G	(Ib)	101 KIAEAKAIYL DSSPVRSRIE AKKYTTPIRH GSVTYYGPFI FADDHVGGKG	150
SE-SVA-1033-3F	(Ib)	101 KIAEAKAIYL DSSPVRSRIE AKKYTTPIRH GSVTYYGPFI FADDHVGGKG	150
SE-SVA-1033-9C	(Ib)	101 KIAEAKAIYL DSSPVRSRIE AKKYTTPIRH GSVTYYGPFI FADDHVGGKG	150
4p168	(III)	101 KIAEAKAIYL DSSPVRSRIE AKKYTTPIRH GSVTYYGPFV FADDHVGGKG	150
FA281107	(III)	101 KIAEAKAIYL ESSPVRSRIE AKKYTTPIRH GSVTYYGPFV FADDHVGGKG	150
(NO-2007-50-385)
D111E
G-protein (aa 1-50)
DK-3592B	(Ia)	1 MEWNTFFLVI LIIIIKSTTP QITQRPPVEN ISTYHADWDT PLYTHPSNCR	50
M Rhabdo	(Ib)	1 MEWNTFFLVI LIIIIKSTTP QITQRPPVEN ISTYHADWDT PLYTHPSNCR	50
SE-SVA-14-3D	(Ib)	1 MEWNTFFLVI LIIIIKSTTP QITQRPPVEN ISTYHADWDT PLYTHPSNCR	50
SE-SVA-14-5G	(Ib)	1 MEWNTFFLVI LIIIIKSTTP QITQRPPVEN ISTYHADWDT PLYTHPSNCR	50
SE-SVA-1033-3F	(Ib)	1 MEWNTFFLVI LIIIIKSTTP QITQRPPVEN ISTYHADWDT PLYTHPSNCR	50
SE-SVA-1033-9C	(Ib)	1 MEWNTFFLVI LIIIIKSTTP QITQRPPVEN ISTYHADWDT PLYTHPSNCR	50
4p101	(III)	1 MEWNTFFLVI LIILIKSTTS QITQRPPTEN ISTYHADWDT PLYTHPANCR	50
4p168	(III)	1 MEWNTFFLVI LIILIKSTTS QITQRPPIEN ISTYHADWDT PLYTHPSNCR	50
NO-2007-50-385	(III)	1 MEWNTFFLVI LIILIKSTTS QITQRPPIEN ISTYHADWDT PLYTHPANCR	50
S47A

## Slide 3
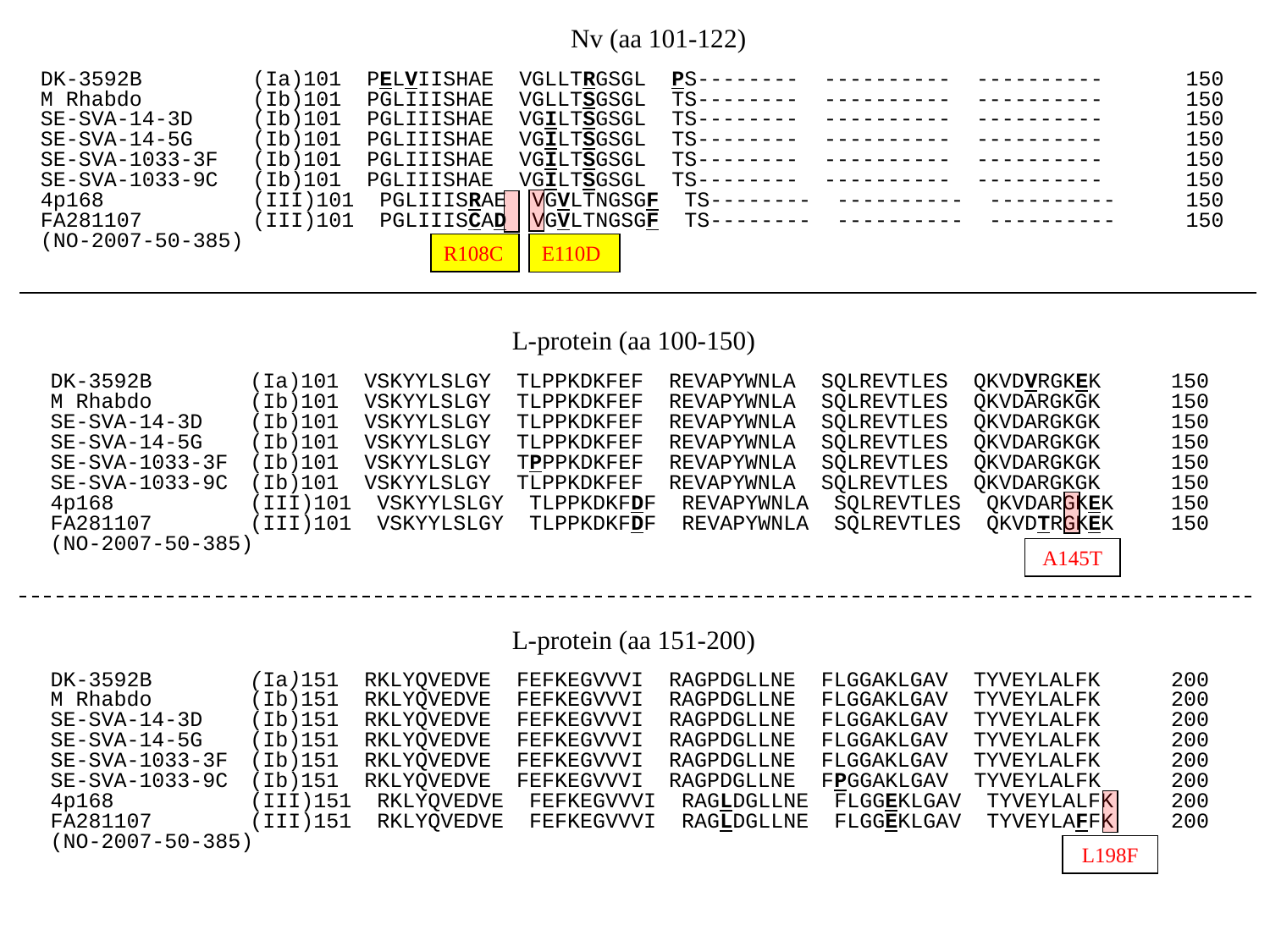

Nv (aa 101-122)
DK-3592B	(Ia)	101 PELVIISHAE VGLLTRGSGL PS-------- ---------- ----------	150
M Rhabdo	(Ib)	101 PGLIIISHAE VGLLTSGSGL TS-------- ---------- ----------	150
SE-SVA-14-3D	(Ib)	101 PGLIIISHAE VGILTSGSGL TS-------- ---------- ----------	150
SE-SVA-14-5G	(Ib)	101 PGLIIISHAE VGILTSGSGL TS-------- ---------- ----------	150
SE-SVA-1033-3F	(Ib)	101 PGLIIISHAE VGILTSGSGL TS-------- ---------- ----------	150
SE-SVA-1033-9C	(Ib)	101 PGLIIISHAE VGILTSGSGL TS-------- ---------- ----------	150
4p168	(III)	101 PGLIIISRAE VGVLTNGSGF TS-------- ---------- ----------	150
FA281107	(III)	101 PGLIIISCAD VGVLTNGSGF TS-------- ---------- ----------	150
(NO-2007-50-385)
R108C
E110D
L-protein (aa 100-150)
DK-3592B	(Ia)	101 VSKYYLSLGY TLPPKDKFEF REVAPYWNLA SQLREVTLES QKVDVRGKEK	150
M Rhabdo	(Ib)	101 VSKYYLSLGY TLPPKDKFEF REVAPYWNLA SQLREVTLES QKVDARGKGK	150
SE-SVA-14-3D	(Ib)	101 VSKYYLSLGY TLPPKDKFEF REVAPYWNLA SQLREVTLES QKVDARGKGK	150
SE-SVA-14-5G	(Ib)	101 VSKYYLSLGY TLPPKDKFEF REVAPYWNLA SQLREVTLES QKVDARGKGK	150
SE-SVA-1033-3F	(Ib)	101 VSKYYLSLGY TPPPKDKFEF REVAPYWNLA SQLREVTLES QKVDARGKGK	150
SE-SVA-1033-9C	(Ib)	101 VSKYYLSLGY TLPPKDKFEF REVAPYWNLA SQLREVTLES QKVDARGKGK	150
4p168	(III)	101 VSKYYLSLGY TLPPKDKFDF REVAPYWNLA SQLREVTLES QKVDARGKEK	150
FA281107	(III)	101 VSKYYLSLGY TLPPKDKFDF REVAPYWNLA SQLREVTLES QKVDTRGKEK	150
(NO-2007-50-385)
A145T
L-protein (aa 151-200)
DK-3592B	(Ia)	151 RKLYQVEDVE FEFKEGVVVI RAGPDGLLNE FLGGAKLGAV TYVEYLALFK	200
M Rhabdo	(Ib)	151 RKLYQVEDVE FEFKEGVVVI RAGPDGLLNE FLGGAKLGAV TYVEYLALFK	200
SE-SVA-14-3D	(Ib)	151 RKLYQVEDVE FEFKEGVVVI RAGPDGLLNE FLGGAKLGAV TYVEYLALFK	200
SE-SVA-14-5G	(Ib)	151 RKLYQVEDVE FEFKEGVVVI RAGPDGLLNE FLGGAKLGAV TYVEYLALFK	200
SE-SVA-1033-3F	(Ib)	151 RKLYQVEDVE FEFKEGVVVI RAGPDGLLNE FLGGAKLGAV TYVEYLALFK	200
SE-SVA-1033-9C	(Ib)	151 RKLYQVEDVE FEFKEGVVVI RAGPDGLLNE FPGGAKLGAV TYVEYLALFK	200
4p168	(III)	151 RKLYQVEDVE FEFKEGVVVI RAGLDGLLNE FLGGEKLGAV TYVEYLALFK	200
FA281107	(III)	151 RKLYQVEDVE FEFKEGVVVI RAGLDGLLNE FLGGEKLGAV TYVEYLAFFK	200
(NO-2007-50-385)
L198F

## Slide 4
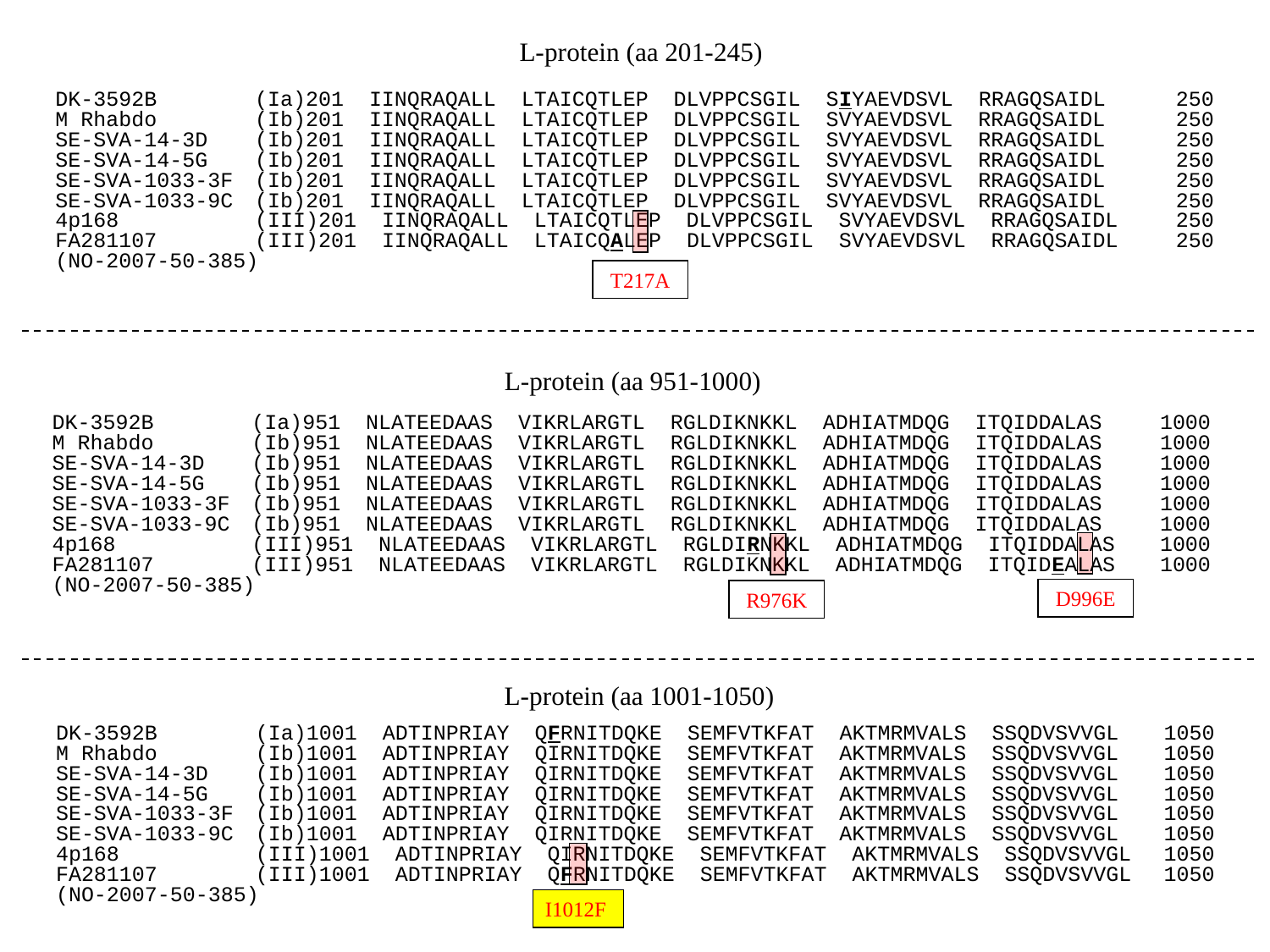

L-protein (aa 201-245)
DK-3592B	(Ia)	201 IINQRAQALL LTAICQTLEP DLVPPCSGIL SIYAEVDSVL RRAGQSAIDL	250
M Rhabdo	(Ib)	201 IINQRAQALL LTAICQTLEP DLVPPCSGIL SVYAEVDSVL RRAGQSAIDL	250
SE-SVA-14-3D	(Ib)	201 IINQRAQALL LTAICQTLEP DLVPPCSGIL SVYAEVDSVL RRAGQSAIDL	250
SE-SVA-14-5G	(Ib)	201 IINQRAQALL LTAICQTLEP DLVPPCSGIL SVYAEVDSVL RRAGQSAIDL	250
SE-SVA-1033-3F	(Ib)	201 IINQRAQALL LTAICQTLEP DLVPPCSGIL SVYAEVDSVL RRAGQSAIDL	250
SE-SVA-1033-9C	(Ib)	201 IINQRAQALL LTAICQTLEP DLVPPCSGIL SVYAEVDSVL RRAGQSAIDL	250
4p168	(III)	201 IINQRAQALL LTAICQTLEP DLVPPCSGIL SVYAEVDSVL RRAGQSAIDL	250
FA281107	(III)	201 IINQRAQALL LTAICQALEP DLVPPCSGIL SVYAEVDSVL RRAGQSAIDL	250
(NO-2007-50-385)
T217A
L-protein (aa 951-1000)
DK-3592B	(Ia)	951 NLATEEDAAS VIKRLARGTL RGLDIKNKKL ADHIATMDQG ITQIDDALAS	1000
M Rhabdo	(Ib)	951 NLATEEDAAS VIKRLARGTL RGLDIKNKKL ADHIATMDQG ITQIDDALAS	1000
SE-SVA-14-3D	(Ib)	951 NLATEEDAAS VIKRLARGTL RGLDIKNKKL ADHIATMDQG ITQIDDALAS	1000
SE-SVA-14-5G	(Ib)	951 NLATEEDAAS VIKRLARGTL RGLDIKNKKL ADHIATMDQG ITQIDDALAS	1000
SE-SVA-1033-3F	(Ib)	951 NLATEEDAAS VIKRLARGTL RGLDIKNKKL ADHIATMDQG ITQIDDALAS	1000
SE-SVA-1033-9C	(Ib)	951 NLATEEDAAS VIKRLARGTL RGLDIKNKKL ADHIATMDQG ITQIDDALAS	1000
4p168	(III)	951 NLATEEDAAS VIKRLARGTL RGLDIRNKKL ADHIATMDQG ITQIDDALAS	1000
FA281107	(III)	951 NLATEEDAAS VIKRLARGTL RGLDIKNKKL ADHIATMDQG ITQIDEALAS	1000
(NO-2007-50-385)
D996E
R976K
L-protein (aa 1001-1050)
DK-3592B	(Ia)	1001 ADTINPRIAY QFRNITDQKE SEMFVTKFAT AKTMRMVALS SSQDVSVVGL	1050
M Rhabdo	(Ib)	1001 ADTINPRIAY QIRNITDQKE SEMFVTKFAT AKTMRMVALS SSQDVSVVGL	1050
SE-SVA-14-3D	(Ib)	1001 ADTINPRIAY QIRNITDQKE SEMFVTKFAT AKTMRMVALS SSQDVSVVGL	1050
SE-SVA-14-5G	(Ib)	1001 ADTINPRIAY QIRNITDQKE SEMFVTKFAT AKTMRMVALS SSQDVSVVGL	1050
SE-SVA-1033-3F	(Ib)	1001 ADTINPRIAY QIRNITDQKE SEMFVTKFAT AKTMRMVALS SSQDVSVVGL	1050
SE-SVA-1033-9C	(Ib)	1001 ADTINPRIAY QIRNITDQKE SEMFVTKFAT AKTMRMVALS SSQDVSVVGL	1050
4p168	(III)	1001 ADTINPRIAY QIRNITDQKE SEMFVTKFAT AKTMRMVALS SSQDVSVVGL	1050
FA281107	(III)	1001 ADTINPRIAY QFRNITDQKE SEMFVTKFAT AKTMRMVALS SSQDVSVVGL	1050
(NO-2007-50-385)
I1012F

## Slide 5
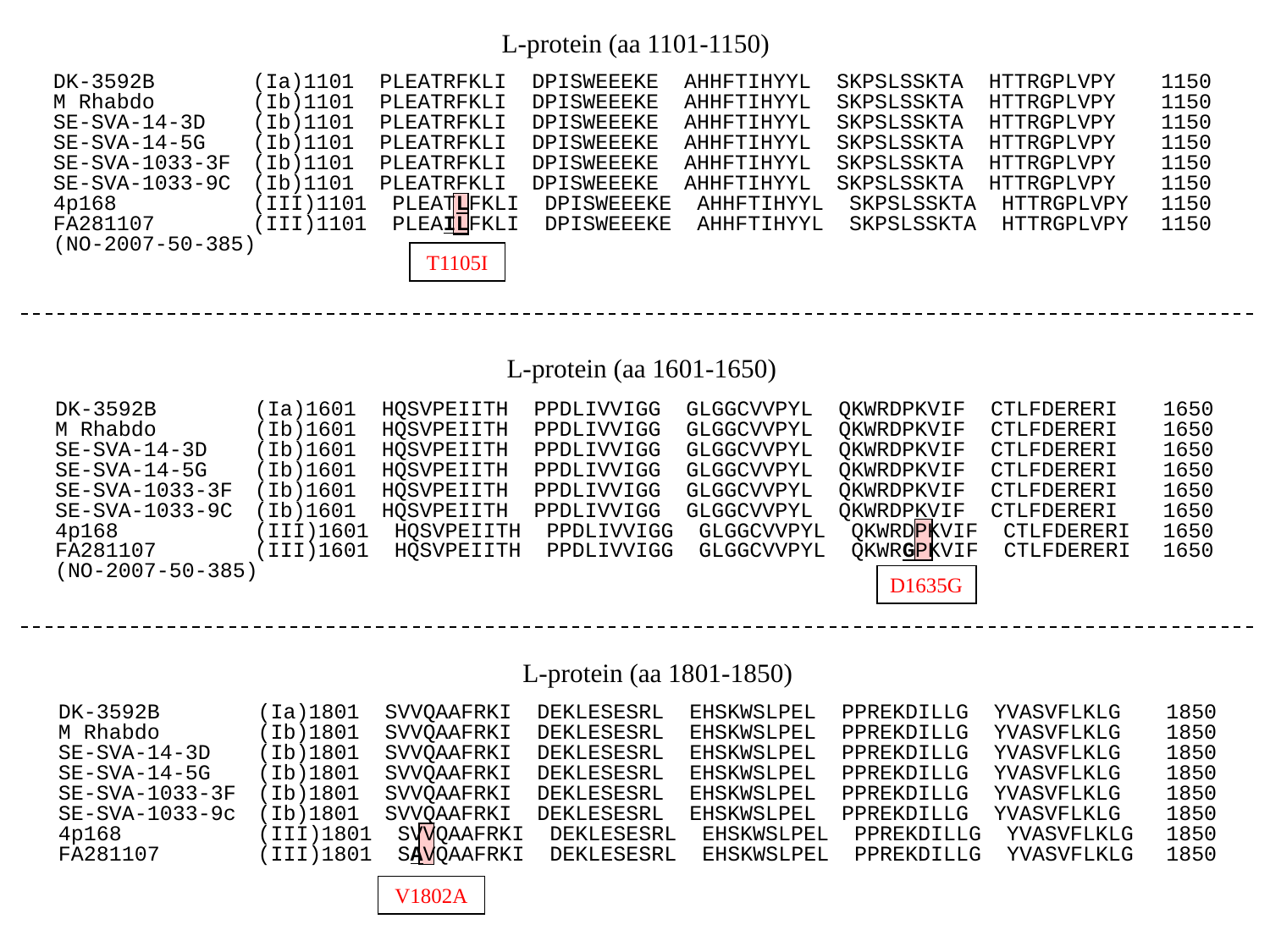

L-protein (aa 1101-1150)
DK-3592B	(Ia)	1101 PLEATRFKLI DPISWEEEKE AHHFTIHYYL SKPSLSSKTA HTTRGPLVPY	1150
M Rhabdo	(Ib)	1101 PLEATRFKLI DPISWEEEKE AHHFTIHYYL SKPSLSSKTA HTTRGPLVPY	1150
SE-SVA-14-3D	(Ib)	1101 PLEATRFKLI DPISWEEEKE AHHFTIHYYL SKPSLSSKTA HTTRGPLVPY	1150
SE-SVA-14-5G	(Ib)	1101 PLEATRFKLI DPISWEEEKE AHHFTIHYYL SKPSLSSKTA HTTRGPLVPY	1150
SE-SVA-1033-3F	(Ib)	1101 PLEATRFKLI DPISWEEEKE AHHFTIHYYL SKPSLSSKTA HTTRGPLVPY	1150
SE-SVA-1033-9C	(Ib)	1101 PLEATRFKLI DPISWEEEKE AHHFTIHYYL SKPSLSSKTA HTTRGPLVPY	1150
4p168	(III)	1101 PLEATLFKLI DPISWEEEKE AHHFTIHYYL SKPSLSSKTA HTTRGPLVPY	1150
FA281107	(III)	1101 PLEAILFKLI DPISWEEEKE AHHFTIHYYL SKPSLSSKTA HTTRGPLVPY	1150
(NO-2007-50-385)
T1105I
L-protein (aa 1601-1650)
DK-3592B	(Ia)	1601 HQSVPEIITH PPDLIVVIGG GLGGCVVPYL QKWRDPKVIF CTLFDERERI	1650
M Rhabdo	(Ib)	1601 HQSVPEIITH PPDLIVVIGG GLGGCVVPYL QKWRDPKVIF CTLFDERERI	1650
SE-SVA-14-3D	(Ib)	1601 HQSVPEIITH PPDLIVVIGG GLGGCVVPYL QKWRDPKVIF CTLFDERERI	1650
SE-SVA-14-5G	(Ib)	1601 HQSVPEIITH PPDLIVVIGG GLGGCVVPYL QKWRDPKVIF CTLFDERERI	1650
SE-SVA-1033-3F	(Ib)	1601 HQSVPEIITH PPDLIVVIGG GLGGCVVPYL QKWRDPKVIF CTLFDERERI	1650
SE-SVA-1033-9C	(Ib)	1601 HQSVPEIITH PPDLIVVIGG GLGGCVVPYL QKWRDPKVIF CTLFDERERI	1650
4p168	(III)	1601 HQSVPEIITH PPDLIVVIGG GLGGCVVPYL QKWRDPKVIF CTLFDERERI	1650
FA281107	(III)	1601 HQSVPEIITH PPDLIVVIGG GLGGCVVPYL QKWRGPKVIF CTLFDERERI	1650
(NO-2007-50-385)
D1635G
L-protein (aa 1801-1850)
DK-3592B	(Ia)	1801 SVVQAAFRKI DEKLESESRL EHSKWSLPEL PPREKDILLG YVASVFLKLG	1850
M Rhabdo	(Ib)	1801 SVVQAAFRKI DEKLESESRL EHSKWSLPEL PPREKDILLG YVASVFLKLG	1850
SE-SVA-14-3D	(Ib)	1801 SVVQAAFRKI DEKLESESRL EHSKWSLPEL PPREKDILLG YVASVFLKLG	1850
SE-SVA-14-5G	(Ib)	1801 SVVQAAFRKI DEKLESESRL EHSKWSLPEL PPREKDILLG YVASVFLKLG	1850
SE-SVA-1033-3F	(Ib)	1801 SVVQAAFRKI DEKLESESRL EHSKWSLPEL PPREKDILLG YVASVFLKLG	1850
SE-SVA-1033-9c	(Ib)	1801 SVVQAAFRKI DEKLESESRL EHSKWSLPEL PPREKDILLG YVASVFLKLG	1850
4p168	(III)	1801 SVVQAAFRKI DEKLESESRL EHSKWSLPEL PPREKDILLG YVASVFLKLG	1850
FA281107	(III)	1801 SAVQAAFRKI DEKLESESRL EHSKWSLPEL PPREKDILLG YVASVFLKLG	1850
V1802A
